# Active mesh and neural network pipeline for cell aggregate segmentation

**DOI:** 10.1016/j.bpj.2023.03.038

**Published:** 2023-03-30

**Authors:** Matthew B. Smith, Hugh Sparks, Jorge Almagro, Agathe Chaigne, Axel Behrens, Chris Dunsby, Guillaume Salbreux

**Affiliations:** 1 The Francis Crick Institute, London, United Kingdom; 2 Photonics Group, Department of Physics, Imperial College London, London, United Kingdom; 3 Cell Biology, Neurobiology and Biophysics, Department of Biology, Faculty of Science, Utrecht University, Utrecht, the Netherlands; 4 Cancer Stem Cell Team, The Institute of Cancer Research, London, United Kingdom; 5 Department of Genetics and Evolution, Geneva, Switzerland

## Abstract

Segmenting cells within cellular aggregates in 3D is a growing challenge in cell biology due to improvements in capacity and accuracy of microscopy techniques. Here, we describe a pipeline to segment images of cell aggregates in 3D. The pipeline combines neural network segmentations with active meshes. We apply our segmentation method to cultured mouse mammary gland organoids imaged over 24 h with oblique plane microscopy, a high-throughput light-sheet fluorescence microscopy technique. We show that our method can also be applied to images of mouse embryonic stem cells imaged with a spinning disc microscope. We segment individual cells based on nuclei and cell membrane fluorescent markers, and track cells over time. We describe metrics to quantify the quality of the automated segmentation. Our segmentation pipeline involves a Fiji plugin that implements active mesh deformation and allows a user to create training data, automatically obtain segmentation meshes from original image data or neural network prediction, and manually curate segmentation data to identify and correct mistakes. Our active meshes-based approach facilitates segmentation postprocessing, correction, and integration with neural network prediction.

## Significance

In vitro culture of organ-like structures derived from stem cells, so-called organoids, allows us to image tissue morphogenetic processes with high temporal and spatial resolution. Three-dimensional segmentation of cell shape in time-lapse videos of these developing organoids is, however, a significant challenge. In this work, we propose an image analysis pipeline for cell aggregates that combines deep learning with active contour segmentations. This combination offers a flexible and efficient way to segment three-dimensional cell images, which we illustrate with segmenting data sets of growing mammary gland organoids and mouse embryonic stem cells.

## Introduction

We describe here a full pipeline for segmenting microscopy images of cells in three-dimensional (3D), using active meshes and artificial neural networks. This includes a plugin for Fiji, Deforming Mesh 3D (DM3D), which provides an assisted way to segment cells in 3D over time. We apply our pipeline to segmentation of dynamic, relatively small cell aggregates (∼10s of cells).

The field of segmenting and tracking cells and nuclei in 3D microscopy images has experienced numerous recent developments (1). Semiautomated or assisted tools such as ilastik (2) or Labkit (3) can be used to segment images using pixel classification. Leveraging neural networks, techniques such as StarDist (4) allow the users to generate segmentations automatically, in the case of StarDist by localizing nuclei using star-convex polygons. In these tools, segmentations can be obtained by either using a pretrained model, or creating training data manually and training a new model, or by augmenting an existing model through generating new training data and further training. Other tools that use neural networks are Cellpose (5), which creates a topological map where gradient flow tracking (6) is used to find the contour of the cell, and EmbedSeg (7), an embedding-based instance segmentation method. These techniques are appropriate for detecting and segmenting cells as binary blobs. Another technique to segment cells involves creating a mesh representation and evolving active contours to best fit the image (8,9,10). Integrating tracking with detection can improve segmentation efficiency, as tracking algorithms or networks can be used to predict cells in successive frames and improve the seeding of new cells for segmentation (11,12,13,14,15,16).

Our technique uses a workflow common to other neural network-based methods: the user can manually segment a subset of data, then use a neural network to automatically create more segmentations for the remaining data. Our method, however, incorporates the use of active meshes in this workflow for initial manual segmentation, for automatically segmenting the neural network generated images, and for manual correction. This brings an important advantage, as editing meshes in 3D is an intuitive and convenient way to perform 3D segmentation, notably compared with using 2D pixel-based segmentation tools. Active meshes are handled and deformed using a custom-made Fiji plugin, DM3D. This plugin is based on an implementation of an active mesh deformation method and handles several segmentation meshes in the same image frame.

**Figure 1:**
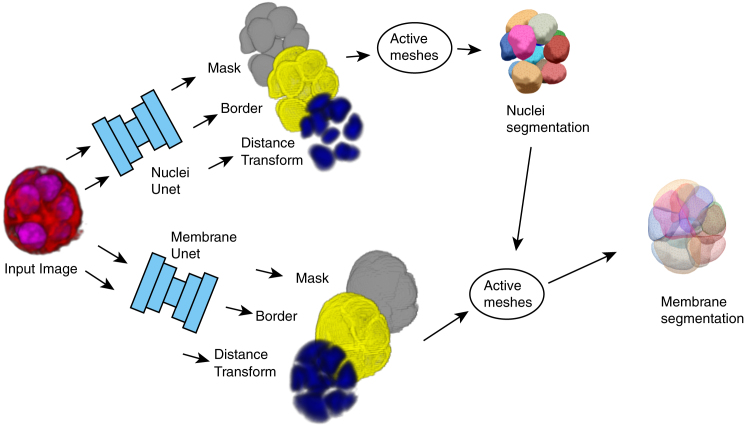
Overview of segmentation pipeline, from an original two-channel 3D fluorescent microscopy image to a set of meshes that represent the cell nuclei and the cell membranes. To see this figure in color, go online. For a Figure360 author presentation of this figure, see https://doi.org/10.1016/j.bpj.2023.03.038.

In our pipeline (Fig. 1), manually obtained 3D meshes are used to create labels that are learned by a neural network with a 3D Unet architecture (17). One of the labels the neural network learns to create is the distance transform, a label that associates to each voxel a value corresponding to its distance to the edge of the object it is associated with. The distance transform or watershed transform (18) have been used previously in combination with deep learning neural networks for object detection and separating overlapping objects (18,19).

The trained neural network processes a 3D time-lapse video and predicts a modified distance transform for each voxel within each frame. The distance transform is modified in the sense that it takes nonzero values only within the surface that it measures the distance from. This distance transform is used to locate 3D regions that represent individual cells or their nuclei. A triangulated mesh is initialized within each of these regions. An active mesh method is then used to deform the mesh to the outer surface of nuclei or cell membranes.

**Figure 2:**
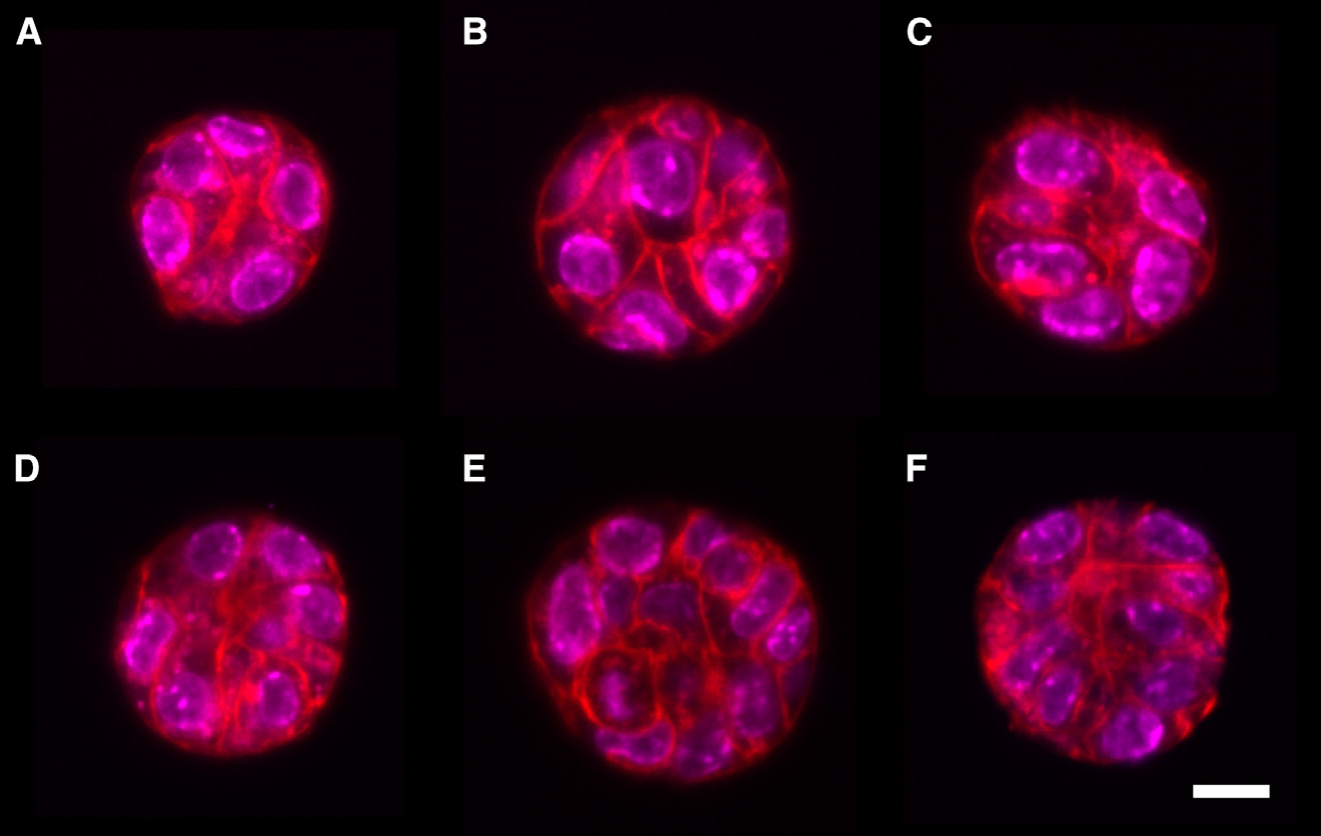
*x*-*y* cross sections through the equator of six different organoids after 8 h of imaging. Scale bar, 10 μm. Red label, membrane dye; magenta, DNA. Organoids in (*A*–*F*) are later referred to as Movies 1–6, corresponding to Video S1. Segmentation result for cell membrane and cell nuclei for Movie 1, view removing centroid motion, Video S2. Segmentation result for cell membrane and cell nuclei for Movie 2, view removing centroid motion, Video S3. Segmentation result for cell membrane and cell nuclei for Movie 3, view removing centroid motion, Video S4. Segmentation result for cell membrane and cell nuclei for Movie 4, view removing centroid motion, Video S5. Segmentation result for cell membrane and cell nuclei for Movie 5, view removing centroid motion, Video S6. Segmentation result for cell membrane and cell nuclei for Movie 6, view removing centroid motion. To see this figure in color, go online.

To demonstrate the effectiveness of our technique we segmented and tracked six mammary gland organoids for 24 h at 11 min imaging intervals (Fig. 2). Organoids have nuclei labeled with the dye SiR-DNA and membrane labeled with tdTomato (see material and methods). Image data were obtained using multichannel dual-view oblique plane microscopy (20), and we selected organoids that appeared to have good signal/noise at the beginning of the imaging period. We refer to this data set as Movies 1–6, corresponding to Video S1. Segmentation result for cell membrane and cell nuclei for Movie 1, view removing centroid motion, Video S2. Segmentation result for cell membrane and cell nuclei for Movie 2, view removing centroid motion, Video S3. Segmentation result for cell membrane and cell nuclei for Movie 3, view removing centroid motion, Video S4. Segmentation result for cell membrane and cell nuclei for Movie 4, view removing centroid motion, Video S5. Segmentation result for cell membrane and cell nuclei for Movie 5, view removing centroid motion, Video S6. Segmentation result for cell membrane and cell nuclei for Movie 6, view removing centroid motion.

Video S1. Segmentation result for cell membrane and cell nuclei for Movie 1, view removing centroid motion

Video S2. Segmentation result for cell membrane and cell nuclei for Movie 2, view removing centroid motion

Video S3. Segmentation result for cell membrane and cell nuclei for Movie 3, view removing centroid motion

Video S4. Segmentation result for cell membrane and cell nuclei for Movie 4, view removing centroid motion

Video S5. Segmentation result for cell membrane and cell nuclei for Movie 5, view removing centroid motion

Video S6. Segmentation result for cell membrane and cell nuclei for Movie 6, view removing centroid motion

To segment this data set, we first generated original training data by manually creating segmentations of a subset of the data. We then processed the whole data set with a trained neural network to obtain initialization for segmentation meshes, which are deformed using the DM3D plugin. We then refined the generated segmentations by manual inspection and tracking cells with DM3D, to segment the complete time-lapse videos.

To evaluate the quality of the neural network segmentations, we prepared a ground truth data set from manual segmentations and compared that with segmentations from the fully automated pipeline. We show an overview of the segmentation results, and a measure of their quality by comparing results from the pipeline with manual segmentations.

To also verify that our pipeline can be applied to different types of cells and microscopy images, we also quantify segmentation results of mouse embryonic stem cells (mESCs) imaged with a spinning disc microscope.

## Methods

### Manual segmentation of original image data

**Figure 3:**
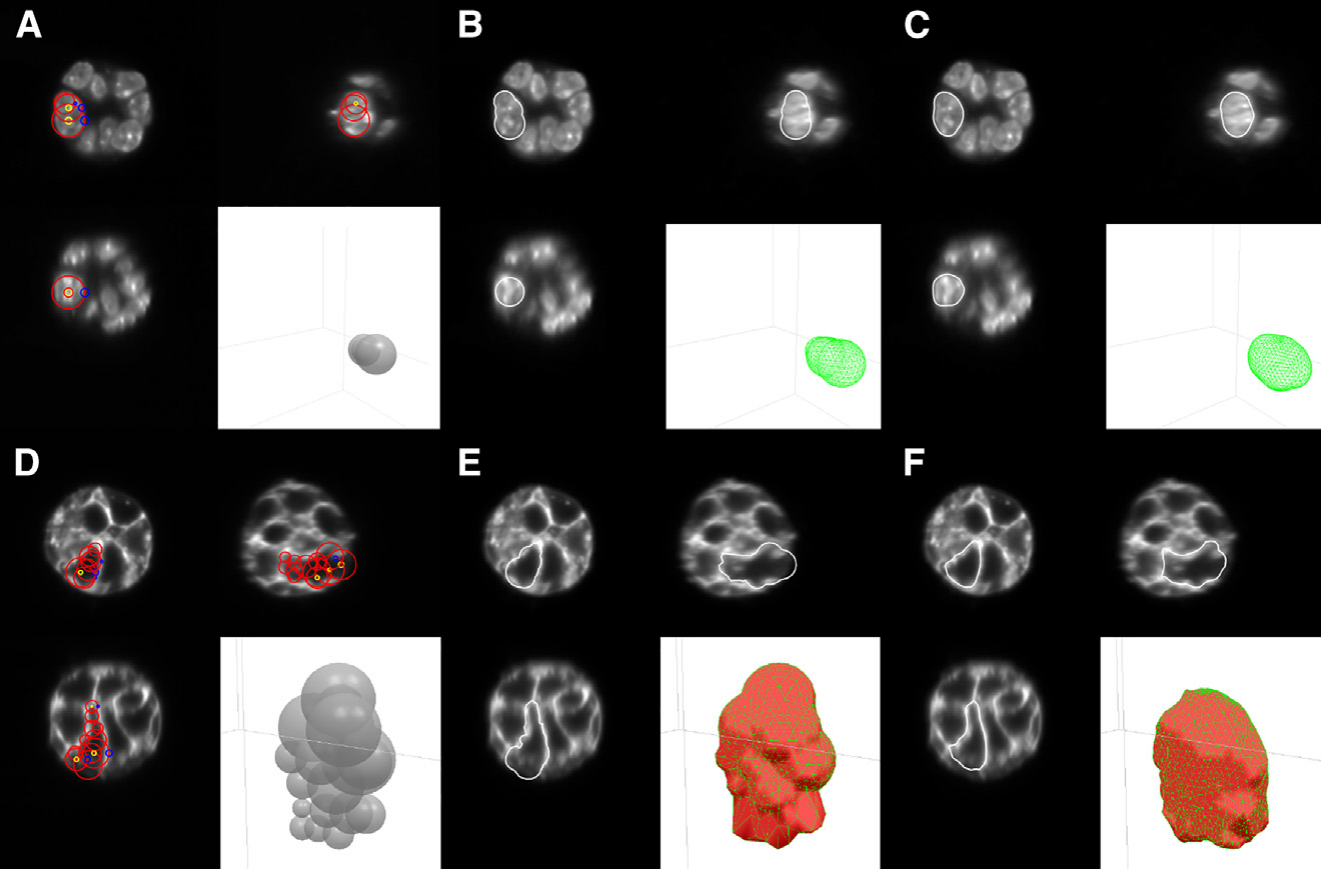
Manual initialization of segmentation meshes that are then deformed using the active mesh method to the cell nucleus (*A*–*C*) or to the cell membrane (*D*–*F*). (*A* and *D*) Orthogonal cross section views and a 3D view during mesh initialization. Red circles: boundaries of the spheres used for mesh initialization. The yellow and blue circles are handles that can be manipulated by the user to adjust the position and radius of the spheres. (*B* and *E*) Same orthogonal views with the initialized mesh. (*F* and *G*) Mesh after deformation to the nucleus or cell membrane image intensity. To see this figure in color, go online.

Here, we describe the mesh-based segmentation technique we use to manually segment cell nuclei and cell membranes from original image data (Fig. 3). To generate manual segmentation using DM3D we initialize a coarse version of the nucleus or the cell to segment in 3D. This is performed by manually positioning spheres within the nucleus or the cell, trying to capture their shape. A mesh approximating the shape of the resulting collection of spheres is created, using a raycast technique to fill the spheres (8). This initial mesh is subsequently deformed to conform to the nucleus shape, by minimizing an effective energy with two contributions: an intrinsic force that depends on the mesh shape as described in Appendix B1, and a force arising from an “image energy” that depends on the mesh and on the voxel values. We use different effective energies for manually segmenting nuclei and cell membranes from original image data, as described below.

#### Segmentation of cell nuclei from original image data

To deform meshes to outer surfaces of nuclei, we use a “perpendicular gradient energy.” Labeled nuclei are essentially 3D-filled continuous regions of high intensity. Therefore, we use an energy that is based on the gradient of the nuclear channel (21). We denote I(x) the image intensity at a voxel position x. We associate a unit normal vector n to a node on the mesh by averaging and normalizing the unit normal vectors to triangles connected to the node. The energy associated with a node on the mesh and evaluated at position x is then defined as:

(1) $$E_{img}\left(x\right)=-{\left[\frac{\sum_{i=-w}^{w}k_{i}I\left(x+in\right)}{\sum_{i=-w}^{w}\left|k_{i}\right|}\right]}^{2}\text{,}$$

where we choose w=5, and the coefficients $k_{i}$ are obtained from the derivative of a Gaussian kernel with standard deviation σ:

(2) $$k_{i}=-\frac{i}{\sqrt{2\pi }\sigma^{3}}\;\exp\;\left[-\frac{i^{2}}{2\sigma^{2}}\right]\text{.}$$

Eq. 1 corresponds to an approximate evaluation of the square magnitude of the intensity gradient along the direction n. We choose σ=2 pixels, a value which we determined empirically to ensure high enough smoothing of intensity profiles while maintaining a low computing cost. To obtain a force acting on a mesh node, one evaluates a finite difference:

(3) $$F=-\frac{w_{img}}{2}\left[E_{img}\left(x+n\right)-E_{img}\left(x-n\right)\right]n\text{,}$$

with $w_{img}$ a factor modulating the weight of the contribution of the image energy relative to the intrinsic mesh forces. To calculate the energy at a point not located exactly at the center of a voxel, we use linear interpolation to evaluate the intensity I(x). This force is added to a force contribution intrinsic on the mesh, which depends on its curvature and the distance between nodes to penalize surface bending and surface area (8) (Appendix B1).

#### Segmentation of cell membranes from original image data

To segment the membrane we use a “perpendicular intensity energy.” As the labeled membrane can be considered as a bright surface, we use an energy that attracts a mesh node to regions of high intensity. Considering a node at position x with unit normal vector n, defined as in the previous section, we write:

(4) $$E_{img}\left(x\right)=-\frac{1}{N}\int duG_{\sigma }\left(u\right)I\left(x+un\right)$$

where $G_{\sigma }$ is a 1D Gaussian kernel with standard deviation of 2 pixels, and N is a normalization factor. Eq. 4 corresponds to a convolution operation between the kernel $G_{\sigma }$ and the intensity profile *I* evaluated along the normal n.

We then use the following force acting on a mesh node at position x, obtained by evaluating a discretized version of the gradient of the energy $E_{img}$ in Eq. 4, along the normal to the mesh node n:

(5) $$F=w_{img}\frac{\sum_{i=-w}^{w}k_{i}I\left(x+in\right)}{\sum_{i=-w}^{w}\left|k_{i}\right|}n\text{,}$$

where $k_{i}$ is defined in Eq. 2, and $w_{img}$ is a factor modulating the weight of the contribution of the image energy relative to the intrinsic mesh forces.

Here, one can use a collection of manually created spheres to initialize a segmentation mesh, similar to what was done to segment cell nuclei; alternatively one can also use the nuclear mesh as initialization and subsequently deform it to the membrane channel.

#### Manual improvement of active mesh segmentation

A segmentation problem arises when the mesh does not stabilize to a steady state that suitably follows the contour of the object. Such a situation can be caused by image artifacts, poor initialization, or a poor choice of mesh deformation parameters. These issues can be addressed with DM3D by interactively editing meshes. Meshes can also be manually initialized more closely to the desired shape. Parameters α and β affecting the mesh evolution can be adjusted (see Appendix B1 for a definition), and the resulting effect on mesh deformation can be observed directly within the plugin. Mesh deformation iterations can also be performed by modulating the weight $w_{img}$ of the image energy relative to the intrinsic mesh energy. Reducing the role played by the intrinsic mesh energy allows the mesh to capture more prominent, irregular features of the cell nucleus or membrane. An additional tool is available within the DM3D plugin to manually edit meshes and deform them to the desired output.

### Neural network training

To train the neural network, we initially generated manual segmentations of cell nuclei and membranes for three time frames of Movie 2 (corresponding to Video S8). Manual segmentation meshes are used to create training labels to train a 3D Unet (17). As first described in (22), we modified the Unet architecture to predict three separate labels (Appendix C): 1) a binary mask label that indicates all voxels contained within a mesh, 2) a binary label indicating the border of the binary mask, and 3) a distance transform label with values ranging from 0 to 32. Labels are created for training by first generating a binary image (see Appendix B3) from all cell nuclei meshes or all cell membrane meshes; in this binarization, voxels that are contained within a mesh have value 1, and voxels outside have value 0. This binary image directly provides the mask label, while the binary label for the border are the edge voxels of the mask label. The distance transform is obtained by iteratively eroding the binary image in 3D, and labeling the eroded voxels with the current iteration depth value: the 0th depth eroded corresponds to border voxels, while voxels eroded at the next iteration have distance transform value of 1, and this process is iterated. We choose to saturate the distance transform value to 32, for ease of manipulation of images.

Video S8. Segmentation result for cell membrane and cell nuclei for Movie 2, view removing centroid motion and following solid rotation of the organoid

Two neural networks were trained using labels calculated from the nuclei and membrane meshes, respectively. Each network is trained to learn all three labels simultaneously by using a loss function that is the sum of three loss functions:

(6) $$L=w_{e}L_{e}+w_{k}L_{k}+w_{d}L_{d}\text{,}$$

where $L_{e}$, $L_{k}$, and $L_{d}$ are loss function for the border, mask and distance transform labels respectively, and $w_{e}$, $w_{k}$, and $w_{d}$ are the corresponding weights in the total loss function. $L_{e}$ and $L_{k}$ are Sorensen-Dice coefficient loss functions, L=(|TP|+1)/(|T|+|P|+1), and $L_{d}$ is the log mean-square error $L_{d}=\log\left({\left(T-P\right)}^{2}\right)$, with *T* the truth pixel values and *P* the network predicted pixel value. Neural network parameters can be adjusted to optimize the segmentation results. Here, we found that setting the weights $w_{e}=w_{k}=w_{d}=1$ in Eq. 6 led to acceptable results.

The distance transform contains in principle all of the information of the other two channels, so strictly speaking the membrane and mask channels do not need to be learned by the neural network. However, training the network to learn the membrane and mask labels helps to determine if the network is training properly. Incorrect learning of one of the training labels indeed likely indicates a problem with the training data.

### Obtaining nuclei segmentation meshes

To test the pipeline, we first used the network trained on nuclei labels to obtain nuclei segmentation meshes for all frames of Movie 2. To achieve this, we used the neural network to predict the distance transform of all frames of the videos. The predicted distance transforms are then turned into a binary image through a thresholding step, and continuous regions are labeled and filtered by size. We found that using a distance transform threshold of 1 did not allow to separate all nuclei, as some nuclei are close to each other. To address this, we selected a higher threshold value of 3, and use a region growing or watershed algorithm to expand the detected regions, based on the distance transform image. The detected regions are then used to seed meshes, as follows: for each region, an approximately spherical mesh is generated by creating an isocahedral mesh, centered at the center of mass of the region, and subsequently subdividing the triangles of the mesh. Rays are cast from the center of mass of the region toward nodes of the spherical mesh. Each node is repositioned to the furthest voxel on the inner surface of the detected region that intercepts the corresponding ray (8). The initialized mesh is then deformed by calculating the perpendicular intensity energy of the distance transform with a negative image weight (see segmentation of cell membranes from original image data). This causes the mesh to be attracted to low values of the distance transform, away from the internal volume of the nucleus. A choice of positive and sufficiently large value of the parameter α (Appendix B1) counteracts this effect by ensuring that the mesh tends to shrink and so wraps around the nucleus.

The step of mesh deformation is strongly affected by the quality of the neural network prediction. When the regions detected from the distance transform predicted by the neural network appear to correspond to a visible nucleus, the mesh deformation process reaches a steady state. When a steady state cannot be found by the active mesh deformation algorithm, the mesh tends to shrink and can then be removed after detection of small volume meshes. This can indicate a false positive, where the neural network wrongly identifies a nucleus and the corresponding region needs to be removed. Failure of the mesh to converge to steady state therefore acts as a filtering step.

To evaluate the segmentation results, we plotted the total number of cells over time. Fluctuations in cell count that do not correspond to cell division indicated that the network was failing to accurately segment some frames. For the first video we segmented, Movie 2, a large number of mitosis events were causing the network to fail. We used DM3D to manually segment five additional frames (numbered 21–25) and trained the network using these additional data. After another iteration, we found that the later frames of the video had some degradation in segmentation quality, due to a change in image quality. We therefore manually corrected a late time point (frame 132), and again trained the network including this frame. This step reduced the number of corrections required to segment late time points.

### Obtaining cell membrane segmentation meshes

To obtain cell membrane segmentation meshes, we use the predicted nuclei meshes to initialize active meshes, and deform them to the membrane distance transform predicted by the neural network trained using manually obtained membrane labels. We use a perpendicular intensity energy (Eq. 5) with a negative weight $w_{img}$ to ensure that the mesh is converging to minima of the distance transform.

## Results: Segmenting mammary gland organoids

### Test of fully automated pipeline on seen and unseen data

#### Automated nuclei segmentation

**Figure 4:**
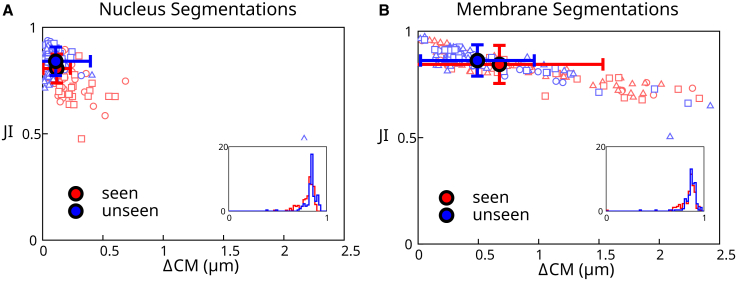
Analysis of automated segmentation quality. (*A* and *B*) Scatterplot of best Jaccard index (JI) versus the distance between the ground truth center of mass and the predicted center of mass (ΔCM) for cells from a “seen” and an “unseen” data set. The *filled circles* represent the mean values of the data points, and the error bars reflect the standard deviation. (*A*) Results of automated segmentation of cell nuclei at full resolution (voxels with side length 0.175 *μ*m). A nucleus diameter is about 8 *μ*m. (*B*) Results of automated segmentation of cell membrane at full resolution. Insets: histogram of best JI distributions. Individual data points outside of the plot range: (*A*) 1/300, (*B*) 2/300. To see this figure in color, go online.

To verify the quality of segmentation results, we compared fully automated segmentations with manually segmented validation data (Fig. 4). We used two sets of validation data: nine “seen” 3D images that correspond to the training data taken from Movie 2 and six “unseen” 3D images which consist of single frames from Movies 1–6 (Fig. 2) that the network has not seen during training. The ground truth is a labeled image generated from manually segmented meshes, where each mesh is binarized and labeled with a unique number. A fully automated segmentation is generated as follows: the neural network is used to create a distance transform image for nuclei. Seed points are then determined from the distance transform based on a thresholding step with a threshold value of 3. Seed points are used to initialize segmentation meshes for nuclei. These segmentation meshes are deformed using the perpendicular intensity energy of the distance transform, as described in obtaining nuclei segmentation meshes. Parameters for mesh iteration are given in Appendix B. The resulting meshes are used to create a fully automated labeled image, which can be compared with the ground truth labels.

To measure the accuracy of the resulting automatic segmentation, we considered two metrics: the best Jaccard index (JI) and the distance between the ground truth and predicted center of mass ΔCM (Fig. 4). The best JI value for cell *i* ${\text{JI}}_{i}$ is calculated for a given ground truth label *i* by calculating the JI between *i* and each prediction label *j*, and finding the optimal value over prediction labels:

(7) $${\text{JI}}_{i}=\max_{j}\left[\frac{T_{i}\cap P_{j}}{T_{i}\cup P_{j}}\right]\text{.}$$

Here, $T_{i}$ denotes the set of voxels with ground truth label *i*, $P_{j}$ the set of voxels with predicted label *j*, $T_{i}\cap P_{j}$ is the size of the intersection between $T_{i}$ and $P_{j}$, in number of voxels, and $T_{i}\cup P_{j}$ the size of the union, in number of voxels. The predicted cell that gives the maximum JI is also used to calculate the distance between predicted and ground truth center of mass, ${\Delta \text{CM}}_{i}$, for cell *i*.

In Fig. 4 *A* we show a scatter plot in the space of values of (${\Delta \text{CM}}_{i}$, ${\text{JI}}_{i}$) for each nucleus, as well as corresponding averages for all detected cells. This graph allows us to visualize the accuracy of nuclei detection and reproduction of their shapes using full resolution images to generate meshes for the nuclei. The pipeline achieves excellent results, with 98% of the unseen segmented cells with a JI above 0.7. Surprisingly, the pipeline achieves overall better results for unseen than from seen data. This may be because some of the seen data set frames were selected because they caused segmentation issues due to cell mitosis or degraded image quality, while the unseen data set was chosen arbitrarily and therefore has no comparable bias.

#### Full resolution images, automated membrane segmentation

We then tested our pipeline on cell membrane segmentation. Here, the automated membrane segmentation was obtained by adjusting meshes obtained from the automated segmentation of nuclei using the predicted distance transform to the cell membrane, as described in obtaining cell membrane segmentation meshes. Parameters for mesh iteration are given in Appendix B. The ground truth segmentation was obtained by manual edits of membrane segmentation meshes. Comparing the result of automated segmentation with the ground truth segmentation (Fig. 4B) shows that the automated segmentation is giving excellent results, although slightly less accurate than nuclei segmentations. This reflects additional difficulty in segmenting cell membranes: their shapes are generally more complex, for instance, due to membrane appendages, which are difficult to identify automatically at the imaging resolution achieved here.

### Full organoid segmentation over time

**Figure 5:**
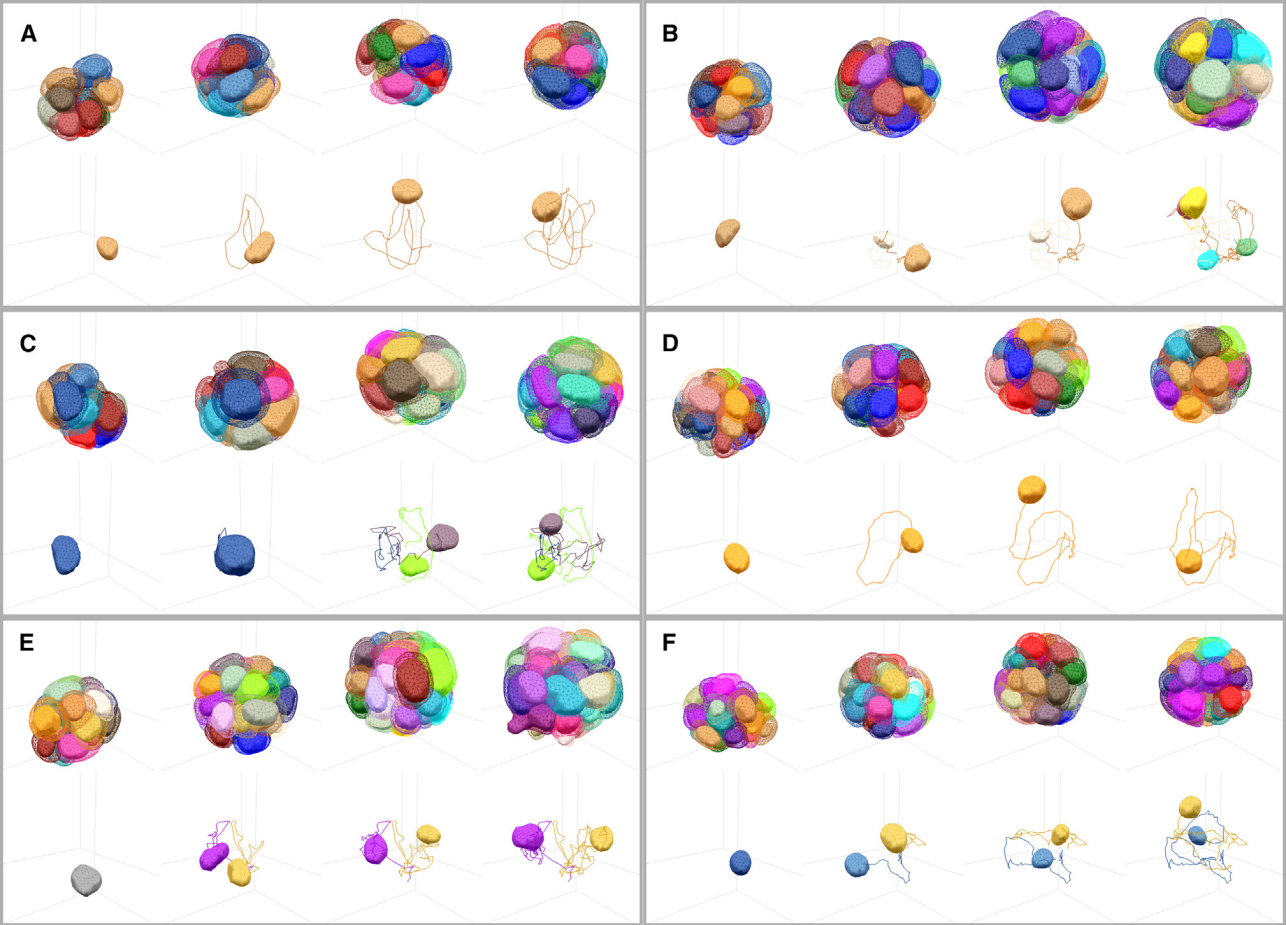
Segmentations results for cell membrane and cell nuclei for six different mammary gland organoids, segmented over 24 h of growth. (*A*–*F*) Within each box, segmentation meshes are shown at 0, 8, 16, and 24 h for each organoid. Within each box, top row: solid volumes correspond to nuclei segmentation meshes and wireframes to cell membrane segmentation meshes. Bottom row: example trajectory of a cell nucleus and the nuclei of the cell progeny during the video. To see this figure in color, go online. See Video S1. Segmentation result for cell membrane and cell nuclei for Movie 1, view removing centroid motion, Video S2. Segmentation result for cell membrane and cell nuclei for Movie 2, view removing centroid motion, Video S3. Segmentation result for cell membrane and cell nuclei for Movie 3, view removing centroid motion, Video S4. Segmentation result for cell membrane and cell nuclei for Movie 4, view removing centroid motion, Video S5. Segmentation result for cell membrane and cell nuclei for Movie 5, view removing centroid motion, Video S6. Segmentation result for cell membrane and cell nuclei for Movie 6, view removing centroid motion, Video S7. Segmentation result for cell membrane and cell nuclei for Movie 1, view removing centroid motion and following solid rotation of the organoid, Video S8. Segmentation result for cell membrane and cell nuclei for Movie 2, view removing centroid motion and following solid rotation of the organoid, Video S9. Segmentation result for cell membrane and cell nuclei for Movie 3, view removing centroid motion and following solid rotation of the organoid, Video S10. Segmentation result for cell membrane and cell nuclei for Movie 4, view removing centroid motion and following solid rotation of the organoid, Video S11. Segmentation result for cell membrane and cell nuclei for Movie 5, view removing centroid motion and following solid rotation of the organoid, Video S12. Segmentation result for cell membrane and cell nuclei for Movie 6, view removing centroid motion and following solid rotation of the organoid. Video S7. Segmentation result for cell membrane and cell nuclei for Movie 1, view removing centroid motion and following solid rotation of the organoid Video S9. Segmentation result for cell membrane and cell nuclei for Movie 3, view removing centroid motion and following solid rotation of the organoid Video S10. Segmentation result for cell membrane and cell nuclei for Movie 4, view removing centroid motion and following solid rotation of the organoid Video S11. Segmentation result for cell membrane and cell nuclei for Movie 5, view removing centroid motion and following solid rotation of the organoid Video S12. Segmentation result for cell membrane and cell nuclei for Movie 6, view removing centroid motion and following solid rotation of the organoid

**Figure 6:**
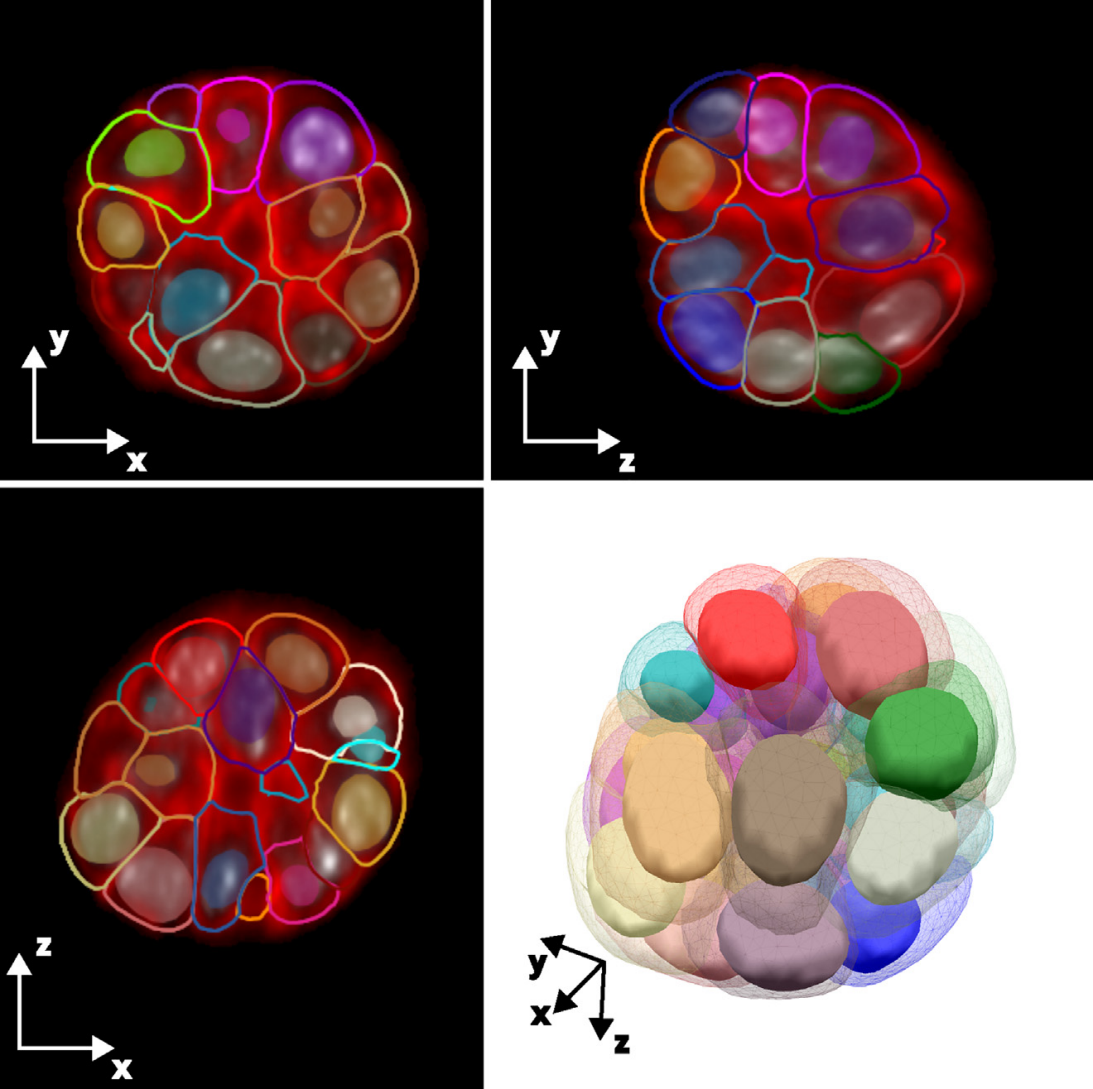
Cross section and 3D view for one frame of one mammary gland organoid shown in Fig. 5. The cross sections display overlay of nuclei (filled volumes) and membrane (wireframes) segmentation meshes on the original data (red, membrane dye; gray, DNA label). To see this figure in color, go online.

We then turned to full segmentation and tracking of the whole 24 h organoid videos (Figs. 5 and 6). Using the automated segmentation steps described in test of a fully automated pipeline on seen and unseen data, we first obtained a fully automated segmentation of nuclei for all six videos.

To compare these results to a ground truth, we then manually corrected them. We proceeded as follows: segmentation of nuclei were used to track the cells over time by using a naive bounding box tracking algorithm (see Appendix B6), and we quantified the cell count over time. Tracking errors and changes in cell count allow to find segmentation errors, when a nucleus appears or disappears, not due to cell division or death. Meshes were corrected by manually initializing a new mesh, deleting incorrect meshes, or splitting meshes that contain multiple nuclei. The corresponding data set constitutes a new ground truth nuclei segmentation.

We then evaluated the detection accuracy of cell nuclei between this manually corrected data set and the automated segmentation, for all time frames in the six organoid videos. To measure the detection accuracy, we mapped predicted to ground truth nuclei. We associate to each nucleus an axis-aligned bounding box, with axis aligned along the *x*, *y*, *z* directions of the image. We then compare the JI values of the bounding boxes of predicted and ground truth nuclei, as defined in Eq. 7. A predicted nucleus maps to the ground truth nucleus in the same frame with the highest JI value. We perform the symmetric operation and map ground truth nuclei to predicted nuclei. If a predicted nucleus and ground truth nucleus are singly mapped to each other, then we count the predicted nucleus as a true positive (TP). When multiple predicted nuclei map to the same ground truth nucleus, then we count those predicted nuclei as false positive (FP). If multiple ground truth nuclei map to a single predicted nucleus, or are not mapped at all, then these ground truth nuclei are counted as false negative (FN). Better networks have a higher number of TP cells, and a smaller number of FP and FN cells. The corresponding results are reported in Table 1. This showed that the automated procedure has an accuracy of ∼90%, as evaluated by the fraction of TP cells.

**Table 1:** Detection accuracy for models at half and full resolution

Model	N	TP	FP	FN	TP/N (%)
Full resolution	18414	16630	182	1637	90.3
Half resolution	18414	18215	72	131	98.9

Data corresponds to frames from all six organoids. *N* corresponds to the total number of segmented nuclei. The “half resolution” model has been trained with 234 additional frames.

**Figure 7:**
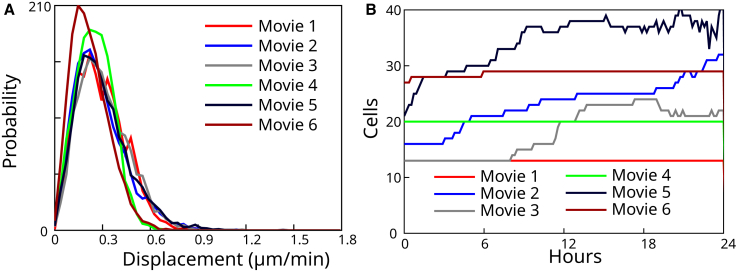
Quantifications associated with tracked nuclei for the six segmented organoids. (*A*) Probability distribution of nucleus velocity, for each individual video. (*B*) Number of cells as a function of time. To see this figure in color, go online.

To visualize the outcome of the full organoid segmentation, we use corrected nuclei segmentation meshes to initialize membrane segmentation meshes. These meshes are then deformed according to a perpendicular intensity energy calculated with the neural network predicted distance transform to cell membranes. Here, the procedure is fully automatic and no further correction is performed. The corresponding results for tracked nuclei and membrane meshes are plotted in Fig. 5. We used these nuclear segmentation results to evaluate cell motion in the organoids. All six organoids are highly dynamic, as quantified by histograms of cell velocity (Fig. 7 *A*). Plotting the number of cells as a function of time also revealed large variations in cell proliferation, with some organoids keeping a constant number of cells while others exhibit a larger number of cell divisions (Fig. 7 *B*).

### Reduction of image resolution and additional training

We then tested if the detection accuracy of cell nuclei could be improved by enlarging the training data set. Incorporating a larger number of full resolution images in neural network training proved to be lengthy; therefore we resorted to half resolution images. Training the network on half resolution images indeed requires eight times less space, memory requirement, and processing time.

To generate training data, we used nuclei segmented meshes from all 134 frames from Movie 2 and 100 frames from Movie 3 (excluding frames which are part of the unseen data set described above), and trained a neural network on images at half resolution. We note that additional ground truth data in this larger data set was manually curated with less accuracy than the original data set used for initial training of the network. The network was trained over 116 epochs, during 10 days on a single Nvidia 3080 GPU workstation. For membrane segmentation data, we used the original training data consisting of 9 frames from Movie 2 at half resolution to train a neural network. Here, the network was trained over 86 epochs, during 9 h on a single Nvidia 3080 GPU workstation.

**Figure 8:**
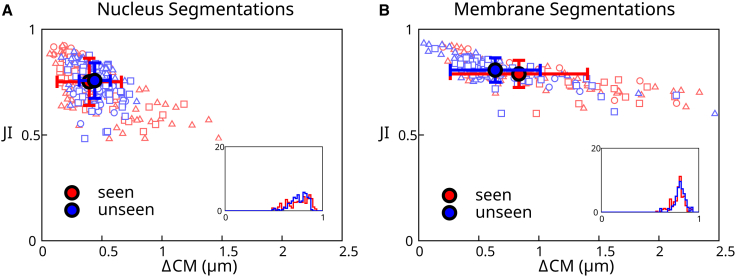
Analysis of automated segmentation quality at half resolution, with a larger training data set. (*A* and *B*) Scatterplot of best JI versus the distance between the ground truth center of mass and the predicted center of mass (ΔCM) for cells from the same “seen” and “unseen” data sets as in Fig. 4 (here the training data set is larger than the seen data set). The *filled circles* represent the mean of the data points, and the errorbars reflect the standard deviation. (*A*) Results of automated segmentation of cell nuclei at half resolution (0.350 *μ*m voxels). (*B*) Results of automated segmentation of cell membrane at half resolution. Insets: histogram of best JI distributions. Individual data points outside of the plot range: (*A*) 0/300, (*B*) 10/300. To see this figure in color, go online.

We then evaluated the quality of mesh segmentation resulting from this newly trained neural network. Comparing Figs. 8 and 4 shows that both the ΔCM prediction accuracy and the JI measurement are slightly worse with decreased image resolution, despite using an enlarged data set. However, the prediction accuracy is still acceptable.

We then evaluated the detection accuracy. Remarkably, training at half resolution with a larger data set increased significantly the detection accuracy, reaching an excellent value of ∼99% (Table 1). We think that this improvement can be attributed to the larger data set used for training. We conclude that half resolution images can be used for efficient and fast nuclei segmentation and tracking, while full resolution images can help with accurate nucleus and membrane segmentation. We note that the mesh representation is based on the actual size of the image volume, so that different scale images can be used with the same set of meshes.

### Comparison to StarDist

**Figure 9:**
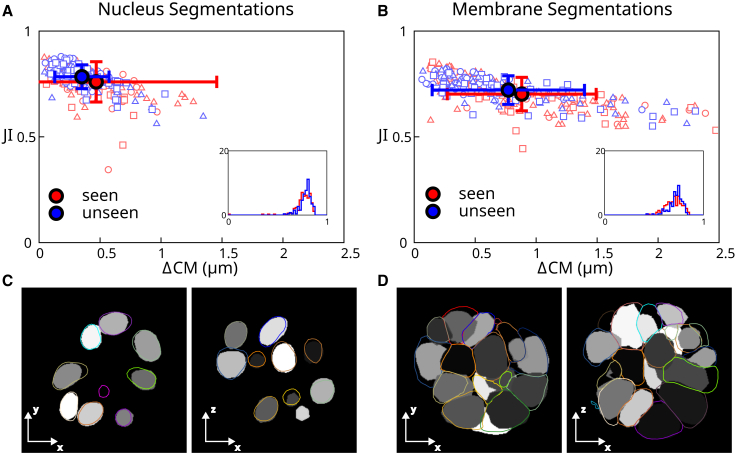
Analysis of segmentation quality with StarDist. (*A* and *B*) Scatterplot of best JI versus the distance between the ground truth center of mass and the predicted center of mass (ΔCM) for cells from a “seen” and an “unseen” data set. The *solid circles* represent the mean value of the data points and the errorbars reflect the standard deviation. (*A*) Results of automated segmentation of cell nuclei. (*B*) Results of automated segmentation of cell membrane. Insets: histogram of best JI distributions. (*C*) Representative example of nucleus prediction from StarDist for two different planes of view. (*D*) Representative example of membrane prediction from StarDist, for two different planes of view. In (*C*) and (*D*), gray regions correspond to StarDist-predicted labels, colored lines indicate ground truth segmentation meshes. Individual data points outside of the plot range: (*A*) 2/300, (*B*) 7/300. To see this figure in color, go online.

We then compared our segmentation results with outcomes obtained from the widely used StarDist software (23). We generated StarDist labels using ground-truths labels from the seen set of images, as described in automated nuclei segmentation. We trained two StarDist models, for the nucleus and membrane labels, respectively, using the default parameters and with full resolution images. We use a provided default parameter of $N_{\text{ray}}=96$ for the number of rays. We then tested the output of StarDist segmentation on the seen and unseen datasets (Fig. 9). We quantified the JI measurement and ΔCM prediction accuracy for nuclei and membrane, as was done using our pipeline (Figs. 4 *A*, *B* and 9 *A*, *B*). The comparison of these quantifications revealed that the StarDist segmentation outcome was slightly inferior to the result obtained with our pipeline for both nucleus and membrane segmentation. However, we cannot exclude that StarDist would not achieve better results by optimizing its parameters. For example, the number of rays determines the level of detail with which StarDist segments objects. We would expect that accurately segmenting cell membranes require more rays than segmenting nuclei. We note that, in any case, a central advantage or our pipeline is the ability to easily manipulate and correct segmentation meshes and use them to generate labels for further neural network training.

### Results: Segmenting aggregates of mESCs

**Figure 10:**
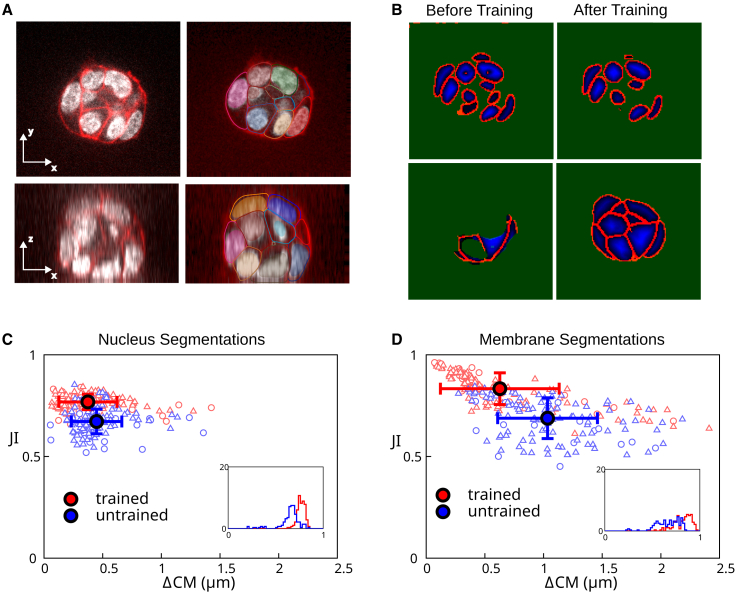
Mouse embryonic stem cell colony imaged on a spinning disc confocal microscope. (*A*) Cross sections of original image (*left*) with ground truth segmentation result overlaid (*right*). White, nuclear label; red, membrane label; other colors, contours of membrane segmentation meshes and filled regions of nuclei segmentation meshes. (*B*) Cross section of neural network output, before and after training the network on the spinning disc images. Top images: nuclei segmentation; bottom images: membrane segmentation. Colors correspond to different outputs of the neural network. Green, mask; red, border; blue, distance transform. Green mask label indicates background. (*C* and *D*) Scatterplot of best JI versus the distance between the ground truth center of mass and the predicted center of mass (ΔCM), before (“untrained”) and after (“trained”) training of the network on two frames of a video of the colony. The *filled circles* are the mean values for the respective datasets and the error bars are one standard deviation.(*C*) Results of automated segmentation of cell nuclei. (*D*) Results of automated segmentation of cell membrane. Insets: histogram of best JI distributions. Individual data points outside of the plot range: (*C*) trained: 0/140; untrained: 10/140; (*D*) trained: 6/140; untrained: 18/140. To see this figure in color, go online.

We then tested our methods on images from a different cell type obtained with a different microscope. We applied our pipeline to a 10-frame video of an aggregate of mESCs imaged with a spinning disc microscope with 5 min time interval between frames (Fig. 10 *A*). The resulting images have nonisotropic voxels, with a pixel size of 244 nm in the *x*-*y* plane and a 2 *μ*m spacing between adjacent slices in the *z* direction. Because our neural network was initially trained on data with isotropic voxels, we interpolated the spinning disc images along the *z* axis to obtain modified images with isotropic voxels of 244 nm. These modified images were then used for training the neural network and segmenting the images.

We first attempted to segment the mESC aggregates with the network previously trained on mammary gland organoid aggregates. We found that the neural network provided outputs that were acceptable for nucleus segmentation, although some border voxels appeared inside the nuclei (Fig. 10 *B*, “before training”). The neural network output for the membrane was, however, strongly underdetecting cells (Fig. 10 *B*, “before training”). To improve on these results, we manually segmented two frames of the video and retrained the neural network. We then generated fully automated segmentation meshes for nuclei and membranes for the 10 frames of the video, as described in the results for mammary gland organoids. We manually corrected these meshes to obtain a ground truth segmentation. We then compared the results of the automated segmentation before and after training the network with two additional frames from the new data set against the ground truth (Fig. 10, *C* and *D*). We found that retraining of the neural network significantly improved the segmentation accuracy, which reached values comparable with our results with mammary gland organoids, despite the limited size of the additional training data set (compare with Figs. 4 and 8). We conclude that we expect that our pipeline can be applied to data sets coming from different microscopes and different cell types.

We observed that mESC aggregates often have closely spaced nuclei, making their segmentation challenging. We found that the ability of the neural network to predict the distance transform aids in separating nuclei from one another, as the predicted distance transform can be used to identify the center of the nuclei.

## Discussion

We showed that using a neural network is an effective way to initialize and deform active meshes on a large number of images. We found that combining active mesh segmentation with a deep learning neural network has several advantages. Notably, active meshes provide a direct and intuitive understanding of the origin of successful or failed segmentation in contrast to neural network predictions. Relaxation of a mesh to a steady state generally indicates that the image is of high enough quality for segmentation to succeed. If the mesh does not reach a steady state, manual inspection of the image helps the user to understand the origin of failure. For instance, in the organoids we have segmented, we have found that automated nucleus segmentation by the neural network could fail because of nuclear dye accumulation artifacts, which could attract the nucleus segmentation mesh, or because of nuclear envelope breakdown during mitosis, as a well-defined nucleus is not visible. Manual mesh initialization and subsequent mesh deformation allows us to correct for these issues. In addition, retraining the neural network after mesh correction allows us to obtain a predicted distance transform which improves on these issues. Overall, the combination of neural network prediction with active meshes allows for efficient manual curation and postprocessing of the segmentation data and improvement of neural network prediction. Following manual curation of segmentation data, retraining of the network improves the outcome of automated segmentation. As Table 1 indicates, we could improve the accuracy in nuclei detection from ∼90 to ∼99% by manually correcting 234 frames and retraining the network, showing the importance of using a neural network in our pipeline. Possibly, repeating these steps of manual correction and network training may allow to further increase this accuracy.

When considering a new data set to segment, manually segmenting with active meshes also allows us to directly test whether the image quality is sufficient for segmentation. This step can be more revealing than directly segmenting a new data set with a neural network, where segmentation failure could arise from inadequate parameters within the neural network, but also from insufficient image quality.

In addition, mesh segmentations are independent of image resolution; this can be useful for locally downloading lower resolution images, or for generating training data at different resolutions.

We also note that the algorithm used to deform the active mesh can be applied to the image directly, instead of the distance transform prediction returned by the neural network. This can in principle ensure that the final segmentation result is independent of the parameters of the neural network and its training history.

Using a neural network also alleviates known drawbacks to active meshes: that an initialization seed has to be found by hand, and that deformation parameters need to be adjusted for different image conditions. Indeed, in addition to providing a high quality initialization of the active mesh, the neural network effectively removes noise and, through the prediction of a distance transform, adjusts signal levels, such that a good set of active mesh parameters will work over a larger range of data qualities.

In this study we have considered two data sets where cells have relatively regular shapes. Our pipeline might have to be adapted to segment more complex cell shapes, possibly by adjusting deformation and remeshing parameters of active meshes.

The DM3D interactive plugin used in this study was built around an active mesh deformation method (8), introduces handling of multiple meshes in the same time frame, steric interaction between meshes, and a remeshing algorithm (Appendix B), so that the plugin is adapted to organoid segmentation. The plugin can also share formats and produce segmentations from a variety of image sources. The plugin also works with virtual image stacks and can be used with or without a 3D display; this makes it practical to work both locally or on remote computers. It is also effective for monitoring segmentations at different points in the pipeline. In an effort to make our plugin more accessible we have added ways to export meshes as other 3D mesh formats, as TrackMate (24) files to apply more advanced tracking algorithms, or as integer labeled images. In addition to describing the DM3D plugin, we report the development of a new 3D-Unet-based segmentation approach that works in conjunction with the DM3D tool. Neural networks and the DM3D plugins are available as described in Appendix A. We provide a tutorial that can be used to analyze example data with six frames and a few cells. Generation of neural network prediction and active mesh evolution take a few minutes on a standard laptop to generate for images used in this tutorial.

We believe that the combination of active meshes and neural network offers a flexible and efficient way of segmenting 3D image data, and we hope that our tool will prove valuable for the scientific community.

## Author contributions

M.B.S., H.S., and J.A. performed research with supervision from A.B., C.D., and G.S. M.B.S. developed and applied the segmentation pipeline. J.A. prepared mammary gland organoids and H.S. performed imaging. A.C. acquired the data in Fig. 9. M.B.S. and G.S. wrote the manuscript with inputs from C.D.

## Data Availability

This project is composed of two open source projects available on github: DM3D an interactive plugin for creating and deforming meshes, and ActiveUnetSegmentation a tensorflow implementation of a 3D Unet available at https://github.com/PaluchLabUCL/DeformingMesh3D-plugin and https://github.com/FrancisCrickInstitute/ActiveUnetSegmentation, respectively. DM3D is also distributed as a Fiji plugin by using the Fiji update site, https://sites.imagej.net/Odinsbane. Additional documentation and usage examples can be found at https://franciscrickinstitute.github.io/dm3d-pages/. A detailed tutorial for the DM3D plugin, with example data, can be found at https://franciscrickinstitute.github.io/dm3d-pages/tutorial.html. Additional data and trained neural networks used in this study can be found at https://zenodo.org/record/7544194.

## Appendix B: Details of the DM3D plugin

In this Appendix we provide details of the active mesh DM3D plugin.

### 1. Mesh iteration

As described in (8), a mesh node *i* with position $x_{i}^{t}$ at pseudotime *t* of mesh evolution, evolves according to the following equation:

(B1) $$\begin{array}{ll} \gamma \left(x_{i}^{t+1}-x_{i}^{t}\right)=F_{i}^{t}-\alpha \sum_{{\left\langle j\right\rangle }_{i}}\left(x_{i}^{t+1}-x_{j}^{t+1}\right)-\beta [2\left(n_{i}-3\right)\sum_{{\left\langle j\right\rangle }_{i}}\left(x_{i}^{t+1}-x_{j}^{t+1}\right) & -\sum_{{\left\langle j\right\rangle }_{i}}\sum_{{\left\langle \left\langle k\right\rangle \right\rangle }_{i,j}}\left(2x_{j}^{t+1}-x_{i}^{t+1}-x_{k}^{t+1}\right)]\text{,} \end{array}$$

where ${\left\langle j\right\rangle }_{i}$ denotes the set of nodes *j* directly connected by an edge to node *i*, $n_{i}$ is the number of nearest neighbors of node *i*, and ${\left\langle \left\langle k\right\rangle \right\rangle }_{i,j}$ denotes the set of nodes *k* neighbors of *j* that are not neighbors of *i*. $F_{i}^{t}$ is an additional force that is obtained from Eq. 3 or 5. α and β are mesh evolution parameters that can be adjusted.

For automated segmentation of nuclei based on the predicted distance transform, we use the perpendicular intensity energy with α=1, β=0.1, γ=1000, and $w_{img}=-0.05$, and perform 100 iterations of each mesh. The same parameters were used for automated segmentation of membranes based on the distance transform, except with $w_{img}=-0.1$, 800 iterations of each mesh, and intermediate steps of automatic remeshing with minimum length 0.75 *μ*m and maximal length 1.6 *μ*m.

### 2. Remeshing

We use a remeshing algorithm that splits long edges (larger than a threshold) and remove short edges (smaller than a threshold). This allows meshes to deform in an unconstrained manner. Remeshing is performed by first sorting each edge by length. The longest edge is then split in two and the two adjacent triangles are replaced by four new triangles. The process is iterated going through edges by decreasing order of length.

Once all of the long edges have been split through this process, short edges are removed if a number of conditions are satisfied. One denotes *i* and *j* the nodes connected by the edge. One then finds the sets of neighboring nodes that share an edge with nodes *i* and *j*. If the neighbors of *i* and the neighbors of *j* have exactly 2 nodes in common, denoted *k* and *l*, then the connection is removed if both *k* and *l* have strictly more than 3 edges connected to them. If the two sets of neighbors have 3 nodes or more in common, the edge is not removed. These criteria allow to prevent meshes with problematic topologies. After removal of the edge, a new node replacing nodes *i* and *j* is generated at the midpoint between nodes *i* and *j*; edges previously connected to *i* and *j* are connected to the new node, and duplicate edges are removed.

The remeshing algorithm significantly improves the quality of meshes by allowing them to deform to more exotic shapes with better distributions of triangles.

### 3. Binarizing a mesh

We use the following procedure to obtain a binary image from segmentation meshes, with label 0 indicating voxels outside of segmentation meshes and label 1 indicating voxels inside meshes. For each y,z value in the 3D image we cast a virtual ray through the mesh, going along the *x* axis. As one progresses along the *x* axis of the image, a topological depth is iterated, starting from value 0. When the ray crosses the mesh, the topographical depth increases by 1 if the scalar product between the normal and the unit vector giving the direction of the ray is negative. Indeed, meshes are defined with normal vector of triangles pointing toward the outside. Conversely the depth decreases by 1 if the scalar product is positive. The voxels are then scanned across and are determined to be inside the mesh if they coincide with a region of positive depth, and outside if they coincide with a region of zero or negative depth.

### 4. Modified distance transform

The modified distance transform is found by iteratively eroding the binary representation of a segmentation mesh. A distance transform image is initialized with voxels with value 0. At first, all positive binary voxels that are neighboring a 0 valued voxel in the binary image are allocated a distance transform value of 0. A new eroded binary image is obtained by setting these voxels to 0. All positive binary voxels that are neighboring a 0 value voxel are then allocated a distance transform value of 1, and a new eroded binary image is obtained by setting these voxels to 0. This process is iterated up to a distance transform value of 32; remaining positive binary voxels are allocated a distance transform value of 32.

### 5. Steric energy

To help with semiautomated mesh-based segmentation, we have introduced a steric energy between active meshes. Several meshes can be evolved simultaneously according to Eq. B1, with an additional contribution to the force $F_{i}^{t}$ that minimizes a steric interaction energy. This method can be used to help deform segmentation meshes, when a feature of the image prevents them from deforming properly if unconstrained. The extra contribution to the force $F_{i}^{t}$ is calculated based on the penetration depth of mesh points into neighboring meshes. Here, we have not used this tool for automated segmentations.

### 6. Tracking algorithm

Tracking is performed by bounding box JI detection. The axis-aligned bounding box of each mesh is calculated, and the bounding boxes of successive frames are used to calculate the JI. Cells with the highest JI between successive frames are mapped to each other. After this first pass tracking algorithm is used, manual tracking error correction can be performed. Tracking errors can be found notably from large displacements or tracks ending abruptly.

## Appendix C: Unet modification

We trained a Unet network with the architecture described in (17), with the following modifications: we use three separate convolution output layers instead of a single one. Two output layers for the mask and distance transform are obtained from the network at depth 0, while the output layer predicting the object border is obtained from the network at depth 1. We use different activation functions for the three output layers: sigmoid activation for the mask and border labels, and ReLu activation for the distance transform.

## Appendix D: Material and methods

### 1. Mouse models

Mice were bred and maintained at the Biological Research Facility of the Francis Crick Institute and the Biological Services Unit of the Institute of Cancer Research. MMTV-PyMT, mTmG, and LifeAct-GFP mice were described before (25,26,27). Mice were kept in individually ventilated cages at 21°C and fed ad libitum. Mouse husbandry and euthanasia for tissue collection was performed conforming to the UK Home Office regulation under the Animals (Scientific Procedures) Act 1986 including the Amendment Regulations 2012. Ear biopsies were sampled for genotyping. All mice were culled by cervical dislocation and confirmation of cessation of circulation.

### 2. Organoid culture

Organoids were established from healthy mammary glands of females aged 10–12 weeks. Mice were humanely culled by cervical dislocation and tissue dissected in aseptic conditions. Mammary glands were digested in 30 *μ*g/mL collagenase (Sigma-Aldrich) in DMEM/F12 (Gibco) using a MACS dissociator (Miltenyi) at 37°C for 20 min. Digested tissue was transferred to a 15 mL centrifuge tube and centrifuged at 3000 rpm, supernatant discarded, and pellet resuspended in 1 mL red blood cell lysis buffer (Sigma-Aldrich) for 3 min. DMEM/F12 (9 mL) was used to stop the red blood cell lysis and tubes were centrifuged in the same conditions. Supernatant was discarded and pellet resuspended in 1 mL of 1× trypsin (Gibco) and incubated at 37°C for 3 min. DMEM/F12 (5 mL) with 10% FBS (Gibco) was used to stop the trypsinization reaction and tubes were centrifuged. Supernatant was discarded and pellet resuspended in 5 mL DMEM/F12, passed through a 70 *μ*m filter, and centrifuged again. Supernatant was discarded and pellet resuspended in Matrigel (Corning) and plated in 25 *μ*L domes, one dome per well of a 24-well plate (Costar) for maintenance. Domes were polymerized at 37°C for 30 min and covered in mouse mammary gland organoid medium consisting of 50 ng/mL EGF (Preprotech), 100 ng/mL FGF (Preprotech), 4 *μ*g/mL heparin (Sigma-Aldrich), 1× B27 (Gibco), 1× N2 (Gibco), 1× penicillin/streptomycin (Gibco), 10 mM HEPES (Gibco), and L-glutamine-containing DMEM/F12 (Gibco). Organoids were maintained at 5% CO_2_ and 20% O_2_ at 37°C. For maintenance, weekly organoid splitting was performed by washing the Matrigel dome in 500 *μ*L PBS, digestion in 300 *μ*L TrypLE (Gibco) for 10 min, dilution of TrypLE in 700 *μ*L DMEM/F12, centrifugation at 3000 rpm for 3 min, discarding supernatant, and resuspending pellet of cells in Matrigel. For microscopy, cells were disaggregated in TrypLE as described before, counted using Trypan Blue and an automatic cell counter, resuspended in Matrigel, and plated in a 96-well plate (Cellvis) in a 30 *μ*L disc per well. Matrigel was polymerized for 30 min at 37°C and wells topped up with 200 *μ*L organoid medium containing 3μM SiR-DNA dye (Spirochrome). For position registration, 1:20 TetraSpeck 0.1 *μ*m beads (Thermo Fisher Scientific) were resuspended in Matrigel, plated in 30 *μ*L discs, and topped up with 200 *μ*L organoid medium.

### 3. Dual-view oblique plane microscopy

For time-lapse imaging of multiple organoids in parallel in a multiwell plate format, a dual-view oblique plane microscope (dOPM) was used, which was a modified version of the system reported in (20). In brief, the system is a type of light-sheet fluorescence microscope (28) that employs a single objective for sample illumination and fluorescence detection (29) designed for multiview single-plane illumination microscopy (30). This type of microscope is suitable for fast 3D imaging with low light dose and reduced sample-induced image artifacts, and so can be applied to time-lapse imaging of multiple live organoids in parallel. For this work, the dOPM configuration reported in (20) was modified to operate with a Nikon 1.2 NA 60× water immersion objective as the primary microscope objective, which has a higher numerical aperture than the original design based around a Nikon 1.15 NA 40× water immersion objective (20). The dOPM system was configured to record 3D image data sets in sample space from the perspectives of overlapping views that are rotated by ±35° relative to one another about the optical axis of the primary microscope objective. Organoids were imaged every 11 min for 24 h totaling 135 time points. From the two dOPM view’s perspectives, the acquired 3D image data per time point consisted of optically sectioned images spaced 0.6 *μ*m apart covering a scan range of 90 *μ*m. Each image plane was 450 pixels in width and height and the pixel size in sample space was 0.175 *μ*m in each dimension to cover a field of view of 140 *μ*m in each dimension. The illumination light sheet used had a calculated full width at half-maximum of 3 *μ*m at the waist in the sample plane.

To image fluorescence from a tdTomato-labeled membrane, a 561 nm laser was used for fluorescence excitation and a 600/52 nm (central wavelength/band pass) emission bandpass emission was used for detection. To image fluorescence from nuclear SiR-DNA, a 642 nm laser was used for fluorescence excitation and a 698/70 nm (central wavelength/band pass) emission bandpass emission was used for detection.

For each spectral channel the information from the 3D data set of each dOPM view was combined into a single 3D data set by using a fusion routine in the Multiview fusion plugin available in ImageJ (31). This routine required registration information to correctly coregister the two dOPM views. This information was determined from dOPM data sets of 3D samples of beads suspended in Matrigel, which were included in the multiwell plate assay as discussed in full resolution images, automated membrane segmentation—see (31) for details of the bead-based coregistration method. Following fusing, the 3D data sets were converted to tiff stacks for segmentation.

### 4. Culture and imaging of mouse embryonic stem cells

Mouse embryonic stem cells (E14 cells stably expressing H2B-RFP) (32) were cultured as described in (33) on 0.1% gelatin in PBS (in N2B27+2i-LIF + penicillin and streptomycin, at a controlled density 1.5–$3.0\times 10^{4}$ cells ${\text{cm}}^{-2}$) on Falcon flasks and passaged every other day using Accutase (Sigma-Aldrich, no. A6964). They were kept in 37°C incubators with 7% CO_2_. Cells were regularly tested for mycoplasma.

The culture medium was made in house using a DMEM/F-12, 1:1 mixture (Sigma-Aldrich, no. D6421-6), Neurobasal medium (Life Technologies, no. 21103-049), 2.2 mM L-glutamine, homemade N2 (see below), B27 (Life Technologies, no. 12587010), 3 *μ*M Chiron (Cambridge Bioscience, no. CAY13122), 1 *μ*M PD 0325901 (Sigma-Aldrich, no. PZ0162), LIF (Merck Millipore, no. ESG1107), 0.1 mM β-mercapto-ethanol, and 12.5 *μ*g mL^−1^ insulin zinc (Sigma-Aldrich, no. I9278). The 200× homemade N2 was made using 8.791 mg mL^−1^ apotransferrin (Sigma-Aldrich, no. T1147), 1.688 mg mL^−1^ putrescine (Sigma-Aldrich, no. P5780), 3 *μ*M sodium selenite (Sigma-Aldrich, no. S5261), and 2.08 *μ*g mL^−1^ progesterone (Sigma-Aldrich, no. P8783), 8.8% BSA.

For colony imaging, the cells were plated on 35 mm Ibidi dishes (IBI Scientific, no. 81156) coated with gelatin the day before the experiment, and imaged on a PerkinElmer Ultraview Vox spinning disc (Nikon Ti attached to a Yokogawa CSU-X1 spinning disc scan head) using a C9100-13 Hamamatsu EMCCD camera. Samples were imaged using a 60× water objective (CFI Plan Apochromat with Zeiss Immersol W oil, numerical aperture 1.2). The samples were imaged acquiring a z stack with ΔZ = 2 *μ*m every 5 min.

## Supporting material

Figure360. An Author Presentation of Figure 4
